# Protein and Lipid Content of Milk Extracellular Vesicles: A Comparative Overview

**DOI:** 10.3390/life13020401

**Published:** 2023-02-01

**Authors:** Sandra Buratta, Lorena Urbanelli, Alessia Tognoloni, Raffaella Latella, Giada Cerrotti, Carla Emiliani, Elisabetta Chiaradia

**Affiliations:** 1 Department of Chemistry, Biology and Biotechnology, University of Perugia, 06123 Perugia, Italy; 2 Department of Veterinary Medicine, University of Perugia, 06126 Perugia, Italy

**Keywords:** extracellular vesicle, exosome, milk, proteomics, lipidomics

## Abstract

The characterization of the protein and lipid cargo of milk extracellular vesicles from different mammal species is crucial for understanding their biogenesis and biological functions, as well as for a comprehensive description of the nutritional aspects of animal milk for human diet. In fact, milk EVs have been reported to possess relevant biological effects, but the molecules/biochemical pathways underlying these effects have been poorly investigated. The biochemical characterization is an important initial step for the potential therapeutic and diagnostic use of natural or modified milk EVs. The number of studies analysing the protein and lipid composition of milk EVs is limited compared to that investigating the nucleic acid cargo. Here, we revised the literature regarding the protein and lipid content of milk EVs. Until now, most investigations have shown that the biochemical cargo of EVs is different with respect to that of other milk fractions. In addition, even if these studies derived mostly from bovine and human milk EVs, comparison between milk EVs from different animal species and milk EVs biochemical composition changes due to different factors including lactation stages and health status is also beginning to be reported.

## 1. Introduction

Extracellular vesicles (EVs) are membrane-surrounded nanoparticles that have been isolated from every fluid of the body, i.e., blood, urine, saliva, amniotic, ascitic, synovial and cerebrospinal fluid, and milk, as well as from the growth medium of cultured cells. These particles are highly heterogenous in size, ranging from 30 to 5000 nm [1]. They were initially identified as a tool to get rid of unnecessary cell content during reticulocytes differentiation [2], but later it was found that they not only contribute to cell homeostasis, but also have a role in mediating cell-to-cell communication, delivering their cargo to recipient cells [3]. The EV content is complex, as they contain not only lipids but also proteins and noteworthy different types of nucleic acids, such as miRNA, lncRNA, mRNA, other small non-coding RNAs [4], and DNA [5]. However, the cargo of EVs is strictly dependent on the releasing cell type and EV subtype [6].

Currently, there is evidence that cells release different populations of EVs that can be classified into three main groups according to their biogenesis and size: exosomes, microvesicles, and apoptotic bodies [7]. However, the field is in continuous evolution and other types of particles are emerging, such as exomeres, i.e., non-membranous nanovesicles with a size ≤50 nm [8], and large oncosomes, i.e., 1–10 μm diameter cancer-derived vesicles originating from the shedding of membrane blebs [9]. Exosomes originate from the internal budding of late endosomal membrane. Consequently, these organelles become full of small intraluminal vesicles (ILVs) and therefore are called multivesicular bodies (MVBs). ILVs are released extracellularly upon MVB exocytosis and consequently take the name of “exosomes” [10]. On the other hand, microvesicles originate from the external shedding of plasma membrane; therefore, they are immediately released extracellularly [11]. Apoptotic bodies are produced from apoptotic cells; therefore, they are not “released”, but appear in concomitance with the disassembly of the dying cells into subcellular fragments [12]. In terms of size, exosomes are the smallest EV type, with an average diameter ranging from 30 to 150 nm [13], whereas microvesicles are heterogeneous, ranging from 100 nm to 1 µm in size [1], and apoptotic bodies are the most heterogenous, ranging from 50 to 5000 nm in diameter [12].

The wide overlap in terms of size has made the isolation of different EV classes a challenging task. In fact, for example, the currently most used methods, such as differential ultracentrifugation and size-exclusion chromatography, are based on EV size and allow to separate small vesicles, enriched in exosomes, from medium/large vesicles, enriched in microvesicles, but they do not allow to obtain preparation exclusively containing exosomes or microvesicles [6,14]. In addition, every biological source of EVs has specific features which may facilitate or hamper the separation of EVs from the rest of the fluid.

EVs have been isolated from every fluid of the body, and milk, which is produced by mammary glands, does not represent an exception. In 1980, a pioneering study reported the presence of epithelial cells and cell fragments in milk [15], and since that study, the interest in milk EVs has expanded to more than 100 publications up to 2022 [16]. Nowadays, the presence of EVs has been demonstrated in milk from different sources (humans, cows, goats, camels, and pigs), and has been also investigated in infant milk formula [17]. Interestingly, the content of EVs in infant milk formula was found to be absent [18] or compromised [19], as compared to fresh or skim milk. As many studies have demonstrated in humans the benefit of breastfeeding over infant milk formula in preventing several diseases [20], this alteration of EV content in infant milk formula has suggested a possible role of EVs in mediating the beneficial properties of milk and has raised considerable interest [21]. 

EVs are the not the only nanoparticles which are present in milk, and this feature has made the isolation and characterization of milk EVs more difficult as compared to other biological fluids [22]. Casein micelles are made up of thousands of casein molecules, most of which form stable complexes with nanoclusters of amorphous calcium phosphate [23]. The average size of casein micelles in cows ranges from 50 to 500 nm and may be slightly different in milk from other species, but usually casein micelle largely overlaps EVs in terms of size [24]. Milk also contains milk fat globules (MFG). These are made up of a core composed primarily of triacylglycerols (TAGs) and bud from the endoplasmic reticulum of the mammary gland alveolar epithelial cells as lipid droplets surrounded by a phospholipid monolayer [25]. These droplets are then secreted and during this process are surrounded by a double layer membrane derived from the mammary gland epithelium [26]. The overall structure is therefore assembled in a peculiar phospholipid trilayer [27]. MFG size may be different in different species, reaching the remarkable dimension of about 10 µm, but the smallest MFG are of nanometric size (a few hundred nanometers) and largely overlaps EVs [28].

The consequence of this complex “nanoparticles composition” is that the isolation of EVs from milk is challenging. The removal of MFG is relatively easy. Usually, when milk is centrifuged at low speed (about 2000× *g*), cells pellet at the bottom of the tubes and MFGs remain on the top and can be skimmed [24]. To increase the removal of MFG, the remaining supernatant can also be filtered [17]. After the MFG removal passage, the supernatant still cannot be processed for EV isolation because it is still rich in proteins, namely casein micelles [29]. Therefore, the supernatant usually undergoes one or more additional steps to remove casein micelles. Different methods have been reported to reach this objective. Acid precipitation accelerates casein micelles aggregation, but there is also evidence that EVs can be damaged by the treatment [30]. Chelating agents such as EDTA have also been used to remove calcium and destabilize casein micelle structure, but EVs may be altered [31]. Ultracentrifugation at intermediate speed (20,000 up to 70,000× *g*) can be used to remove casein micelles, but the removal is partial [24]. More recently, chymosin has been used to hydrolyze casein prior to supernatant ultracentrifugation [32], but the consequences on EVs stability need to be evaluated. 

The consequence of these hurdles in the separation of milk EVs is that the definition of their biochemical content is difficult, even though the analysis of EV cargo is fundamental to assess the origin of milk EVs and gain insight into their function. Current evidence suggests that most milk EVs are secreted from mammary gland epithelial cells [33]. Milk also contains immune cells such as lymphocytes and macrophages that can release EVs [34]. From a functional point of view, milk EVs have raised a considerable interest as theranostics, i.e., therapeutic and diagnostic tools [35]. In fact, several studies have reported that milk EVs enhance the intestinal epithelial barrier function by inducing cell proliferation and production of mucin [36]. Moreover, in intestinal inflammatory disorder models, the administration of milk EVs has shown the ability to modulate inflammation [37] and promote anti-viral activity towards several viruses, namely HIV and CMV [38,39]. For this reason, milk EVs have been proposed as therapeutic agents for intestinal inflammation and intestinal tissue repair [37]. In addition, the composition of milk EVs is influenced by the maternal health condition, as it has been reported that several disorders can affect milk EV composition, such as maternal stress, infection, obesity, or diabetes [21]. Therefore, the analysis of milk EVs may represent an additional diagnostic tool also useful for monitoring the well-being of milk-producing animals [40].

To shed light on the role of protein and lipid milk EV content in modulating their function, here, we report the current findings on the proteomic and lipidomic content of milk EVs. Data presented are sometimes discrepant and the terms “exosomes” or EVs are used in different studies to refer to either the same or different types of vesicles. Furthermore, the methods used for both vesicle isolation and proteomic analysis are different; hence, the results may change also depending on the use of new and more sensitive mass spectrometry approach (Table 1). 

## 2. Protein Content of Milk EVs across Different Mammal Species

Proteins represent one of the main components of milk, with high heterogeneity in terms of the number of protein species that can result from alternative splicing, single point mutations, and different post-translational modifications (PTMs) [57]. The human and animal milk proteomes have been analyzed in order to better understand the nutritional value of this important food across the species. In particular, the analysis of different milk fractions evidenced specific protein profiles with various abundant proteins and some other proteins less represented but with very important functions [58]. Indeed, in addition to their nutritional value, milk proteins can have enzymatic and hormonal activity, as well as immunomodulatory effects [59]. Moreover, the interest in farm animal milk is also due to its use in human diet and to the economic value of the dairy product industry. For these reasons and to define the molecular processes related to lactation and the possible changes due to mammary infection, the proteome of the different milk fractions including MFG, skim milk, and milk whey of humans and farm animals has been extensively characterized [58,60,61,62]. The complexity of milk proteome, in part due to the plethora of PTMs (phosphorylation, acetylation, glycosylation, and lipid conjugation) that differently affect the structural and biological properties of proteins, has been related to its digestibility and allergenicity [63]. In contrast, few studies have analyzed the protein cargo of milk exosomes or, in general, of milk EVs, even if the presence of membrane-delimitated particles distinct from MFG were evidenced in skim milk in the late 1970s and later confirmed [57,64,65]. 

In this section, the literature data regarding the protein cargo of human and animal milk EVs will be reviewed. Like other milk fractions, human and bovine milk EVs have been extensively characterized, while data from other animal species are still limited. In addition, different proteomic approaches and different quantitative mass spectrometry (MS) methods have been used, and a large part of information has been obtained by comparing EV proteomic results with specific database such as Vesiclepedia, EVpedia, and ExoCarta or with protein milk databases (Table 1). 

The paucity of studies about the proteomic analysis of milk EVs is surprising as proteins can be part of their bioactive components and can contribute to the various properties of EVs cited above. Indeed, the immunomodulatory function of milk EVs in the development of the infant’s immune system has been proposed in 2007 by Admyre and co-workers [41], who analyzed for the first time the proteome of the exosomes isolated from human breast milk and colostrum. In these vesicles, high levels of mucin-1, MHC di class II, and tetraspanins CD63 and CD81 were found, whereas some typical protein markers of exosomes including MHC class I, CD54, CD40, CD80, and CD86 were scarcely represented or not detectable. MS analysis evidenced also CD36, polymeric-Ig receptor precursor, immunoglobulins, as well as cytosolic proteins and enzymes. Interestingly, the presence of proteins involved in vesicle budding and endocytosis was also evidenced such as ADP-ribosylation factor and testilin [41]. Moreover, the presence of typical milk proteins including lactadherin, butyrophilin, and xanthine oxidase, that had never been detected in EVs derived from other biofluids or secreted by other cell types, was first assessed in both human [41] and bovine milk exosomes [45]. These data were confirmed by other studies using different methods [43,66]. However, Reinhard and co-workers found that the abundance of these proteins in bovine milk exosomes was lower than in MFG (15–30%-fold less) [45]. On the contrary, milk bovine exosome resulted to be enriched in low abundant milk proteins that are under-represented and/or missing in MFG. This first proteomic analysis of the milk bovine exosomes also evidenced a high enrichment in both Rab proteins and annexins that play key roles in vesicle fusion and trafficking events. Moreover, KEGG pathway analysis includes the proteins specifically identified in bovine exosome in endocytosis, regulation of actin cytoskeleton, and tight junction, highlighting their involvement in milk secretion and exosome formation [45]. 

Notably, Reinhardt and co-workers identified more than 2000 protein species in bovine exosome [45]. Further studies, using different approaches, confirmed that EVs are the milk fractions with the highest protein contents [42,66,67]. In particular, a total of 1963 proteins were identified in human milk EVs, of which 198 seem to be specific of milk EVs, because they resulted to be not present in the EV database Vesiclepedia [42]. Moreover, by comparing the proteins detected in milk EVs with proteome of other milk fractions (e.g., whole milk, skim milk, whey milk, MFG, and casein fraction), reported in previously published studies, 1330 proteins resulted to be common [42]. Proteins identified as exclusive of milk EVs resulted to be linked to cell signaling, as well as cell growth and cell maintenance [42]. In another study, the differentially abundant proteins in milk exosome compared to other milk fractions were also associated to ribosome and regulation of actin cytoskeleton [44]. Moreover, GO annotation confirmed the different cell sources of milk EVs as their proteins were associated to breast, mammary gland, and mammary epithelium, as well as to dendritic cell, CD4 T cell, platelet, monocyte, and B cell [42]. The contribution of bovine mammary epithelial cells (BMECs) as a source of milk EVs was confirmed by Zang and co-workers, who compared the proteome of these cells with the protein database of milk exosomes [68]. Briefly, 77 proteins found in both milk EVs and BMECs resulted to be mainly involved in signaling pathways associated with milk biosynthesis and cell proliferation, according to KEGG pathway annotation.

The overlapping between proteome of exosome and other milk fractions was analyzed in silico [66]. By aggregating the proteins reported in 20 proteomic studies, a protein atlas of milk proteins was generated including more than 4500 protein species. Among them, 3139 proteins resulted to be specifically present only in the exosomes and 95 proteins were common in all milk fractions [66]. Milk EVs are usually isolated from skim milk; therefore, as expected, some proteins must be shared among these two fractions. In contrast, MGF contamination in milk EVs could be limited or excluded as they are obtained from the hydrophobic and hydrophilic fractions of whole milk, respectively. These two phases are usually easily separated in the first step of every protocol used for milk fraction preparations.

The protein cargo differs also between EV subtypes, isolated from commercial skimmed, filtered, and pasteurized cow’s milk, by differential centrifugation. In particular, the proteome of fraction 35K (pelleted at 35,000× *g*) and fraction 100 K (pelleted at 100,000× *g*) was compared [48]. The 20 proteins specifically associated with the 35K fraction resulted to be involved in the regulation of translation, proliferation, and cell survival, whereas 40 proteins identified only in the 100 K fraction were related to metabolism, extracellular matrix turnover, and immunity. In particular, five proteins were highly represented in the 35 K fraction (epidermal growth factor receptor substrate 15, phosphoglycerate dehydrogenase, dynactin subunit 2, protein kinase camp-dependent type regulatory subunit beta, and glutaredoxin-3) and five proteins were specifically identified in the 100 K pellet (complement c8 beta chain, c1galt1-specific chaperone 1, cartilage-associated protein, alpha-mannosidase 2×, and procollagen-lysine 2-oxoglutarate 5-dioxygenase 3). Considering that these proteins discriminating EV subtype have different cellular localization, the authors speculated that the EVs present in the two fractions could be derived from different cellular biogenesis [48]. Furthermore, the glycoproteome of bovine milk exosome has also been characterized [69]. A total of 86 glycoproteins were found to be differentially glycosylated in bovine milk exosome and milk whey. In particular, the fucosylated and sialylated proteins were the most abundant in milk exosomes. Bioinformatic analysis evidenced that milk exosome glycoproteins were mainly involved in immune-related pathways, as well as in signal transduction and cell adhesion [69]. Glycoproteins can be important for the cell crosstalk and drug delivery applications, as it has been reported that the different glycoproteome of EV surface can either assist or inhibit their internalization [70]. 

Among the less-investigated animal species, horse milk exosomes were analyzed by using a 2D electrophoresis-based approach [53]. Few protein spots were evidenced that corresponded to different serum protein species, i.e., albumin, lactoferrin, lactadherin, beta-lactoglobulin, xanthine dehydrogenase, and kappa-, beta-, and alpha-S1 caseins. This is probably due to the specific proteomic approach used which evidenced only the most representative proteins, in turn masking the less abundant. The proteomic analysis of porcine milk EVs identified 639 proteins. These were mainly annotated as cytoplasmatic and membrane proteins and resulted to be involved in carbohydrate metabolism, immunity, and disease-related pathways [51].

### 2.1. Changes of Proteome Milk EVs according to Lactation Stages

The proteome of milk EVs changes during different lactation stages, like that of other milk fractions [41,47,49,52]. Colostrum exosomes are significantly enriched in proteins involved in the immune response such as acute phase proteins, antimicrobial peptides, and complement activation proteins [41,44,47]. Moreover, differences between the proteome of bovine milk and colostrum exosomes are more pronounced than those observed comparing the exosome proteome of human milk and colostrum [44]. Interestingly, by comparing the milk exosome proteins from human and bovine colostrum, 22 milk exosome proteins differentially expressed were annotated in the immune system processes. In particular, lactoferrin-like protein and plastin-2 were the most abundant proteins in bovine and human exosomes, respectively. Lactoferrin protects newborns from the development of necrotizing enterocolitis and regulates cell survival, while plastin-2 is involved in leukocyte function and in the defense against bacteria invasion [44]. Exosomes from both colostrum and mature milk showed high abundance of proteins associated with ribosome and regulation of actin cytoskeleton [44], as well as of different integrins, other typical milk proteins, and proteins regulating cell growth and proliferation [47]. Moreover, the proteome of bovine colostrum EVs progressively changes within 72 h after partum, becoming more similar to those from mature milk [47]. Proteomics analysis evidenced also different protein cargo of exosomes from porcine colostrum and mature milk [52]. Among the identified proteins (637), 166 were found to be differentially abundant. The colostrum EV proteins related to the regulation of hemostasis and cellular lipid intake that can be indicative of the process occurring in the adaptation for extrauterine life were highly represented, whereas the most abundant proteins in mature porcine milk EVs were linked to endothelial barrier, endothelial cell development, and establishment or maintenance of apical/basal cells, probably related to the cellular development linked to the transition from colostrum to milk [52]. Furthermore, the changes of the protein cargo of milk EVs occurred also in the late stage of cow lactation. The comparison between proteome of bovine EVs isolated from late-stage lactating cows and the bovine milk EVs proteomic database evidenced a high abundance of proteins involved in the modulation of immune response, gut functions of infants, lipid intake, as well as structural and functional changes occurring in the mammary gland in late lactations [49].

### 2.2. Milk Protein EVs as Putative Biomarkers 

Changes in milk EVs proteome have made milk EVs a potential source of putative biomarkers assessing the mammary gland pathological or physiological status, since the abundance of several proteins (e.g., casein) in the other milk fractions can limit the detection of less represented proteins, even if these can be differentially modulated in pathological conditions [46,50,71]. The comparison between MFG, whey, and EVs isolated from healthy and Staphylococcus aureus infected cows evidenced higher, larger numbers of protein changes in whey and MFG, compared to EVs [46]. On the contrary, the proteome of milk EVs resulted to be significantly modified by bovine leukemia virus infection [50]. Twenty-six proteins were found to be differentially expressed in milk EVs from infected cattle as compared to uninfected cattle. Bioinformatic analysis annotated these proteins in metabolic processes, binding, catalytic activities, cancer-related pathways, and focal adhesion. Thus, the viral oncogenic disease can alter the proteins encapsulated in bovine milk EVs, and these proteins could be putative markers of clinical stages of the viral disease in cattle [50]. Furthermore, milk exosomes seem to be also promising for the early detection of pregnancy. Notably, in whey EVs from pregnant cows, a significant abundance of proteins was found, such as polymeric immunoglobulin receptor, sulfhydryl oxidase, mucin-1, and lymphocyte antigen 96, already described to be up-regulated in other biofluids or tissues of pregnant cows [71].

## 3. Lipid Composition of Milk EVs

The delimitating membranes of EVs are characterized by a peculiar lipid composition, which determines their properties and biological effects. The lipid composition of EVs depends on the type and physiopathological status of releasing cells. Anyway, as common feature EVs are enriched in sphingolipids (i.e., sphingomyelin and ceramide) and glycerophospholipids containing saturated fatty acids, compared to parental cells [72,73,74]. This lipid asset, which resembles that of lipid rafts, accounts for the high rigidity of EV membrane compared to parental cells and is important for their stability in biological fluids [75]. Furthermore, phospholipids forming EV membrane are also precursors of bioactive molecules (i.e., lysophospholipids and eicosanoids) able to mediate in target cells several processes, such as immune signaling and inflammation.

As for the EVs of other origins, data concerning the content and the biological role of lipids carried by milk EVs are limited compared to research based on nucleic acid cargo. It is well known that milk contains bioactive lipids exerting biological effects such as regulation of neonatal intestinal development and protection against intestinal injury/inflammation [76,77]. Thus, even if the beneficial effects of milk bioactive lipids are documented, there is no information allowing to understand if some of these effects are mediated by lipids carried by EVs.

The first information regarding EV lipid content has been reported by Yassin and colleagues, who analyzed the phospholipid composition of dromedary milk EVs during different lactation stages [56]. This study revealed that phosphatidylcholine (PC) is the major phospholipid component of milk EVs, followed by phosphatidylethanolamine (PE) and phosphatidylserine (PS). This phospholipid distribution is consistent during different lactation periods. Human and bovine milk EVs isolated by two different approaches based on size-exclusion chromatography have been used to analyze their lipid content by thin layer chromatography [24]. In this study, the EV lipid asset was compared with that of MFG. Regarding phospholipid classes, both human and bovine milk EVs were enriched in PS and sphingomyelin (SM) at the expense of PC, compared to MFG membrane (MFGM). The analysis of neutral lipids reveals that bovine milk EVs contained very low levels of TAG, whereas no TAGs were detected in the human milk EVs. Thus, this study highlighted that milk EVs from different species, bovine and human, have similar composition, thus suggesting that EVs present in milk have a conserved membrane asset. Notably, milk EVs have a composition similar to EVs isolated from other body fluids (e.g., urine) and from media of mammalian cell cultures (e.g., prostate cancer cells, human fibroblast, and colorectal cancer cells) [72,73,74,78].

Recently, EVs isolated from bovine milk have been characterized to explore their potential use as a delivery tool of locked nucleic acid-antisense oligonucleotides (LNA) [55]. EVs were isolated from raw bovine milk by a protocol that includes differential centrifugation (crude EVs) followed by a density gradient centrifugation step (purified EVs). The lipid profiles of crude EVs were similar to that of purified EVs. In fact, both samples were characterized by high levels of PC, PE, cholesterol (Chol), and PS. Interestingly, EVs were enriched in PS compared to bovine milk, whereas SM was under-represented [79]. In addition, the content of phosphatidylinositol (PI) in bovine milk EVs is higher compared to EVs released by cultured cells [80]. Furthermore, no significant difference in lipid class distribution was observed either between EVs isolated from fresh bovine milk collected in spring or fall, or between LNA-free and loaded EVs [55]. An important piece of information derived from this study is that the additional density gradient purification step removed contaminating proteins but produced an EV fraction similar to crude EVs in terms of membrane phospholipids [55].

The lipid analysis by LC-MS/MS of preterm and term human milk EVs allowed to identify 395 lipids belonging to 15 lipid subclasses, including PC, PS, and PE [54]. Notably, the most abundant lipid molecular species in both samples were PE(18:1/18:1), PC(18:0/18:2), PC(18:1/16:0), PS(18:0/18:1), and PS(18:0/22:6). The comparison between term and preterm EVs revealed significant differences in the levels of 10 lipid species, 6 of which were up-regulated (SM(d15:1/24:4); TAG (18:2/18:2/22:4); PC(P-16:0/20:5); PC(P-18:0/18:4); PC(P-16:0/18:3); and PC(P-18:0/20:5)) and 4 down-regulated in the preterm group (PC(18:0/20:3); PE(24:4/18:0); PE(P-16:0/20:3); and PC(18:0/20:3)) [54]. Since the aim of this study was to explore the role of the lipid cargo of milk EVs in the prevention of necrotizing enterocolitis (NEC), the top 50 identified lipids in the term and preterm groups were submitted to the Ingenuity Pathway Analysis (IPA) software to predict the signaling pathways associated with these lipids possibly underlying the protective effect. This analysis indicates that the top 50 identified lipids were related to the ERK/MAPK pathway. Consistently, term and preterm human milk EVs reduced the expression of pERK in an in vivo model of NEC. Overall, these results suggest that human milk EV lipids may reduce NEC by inhibiting the ERK/MAPK signaling pathway [54].

Data reported in this paragraph indicate that the lipid composition of both human and bovine milk EVs is similar to that of EVs isolated from other biofluids and from cell culture media, and remarkably different from those of MFG present in milk. MFG are unique lipid trilayers, where the internal monolayer originating from the endoplasmic reticulum is covered by a bilayer formed by the mammary cell membrane during the secretion of the globule with the rest of the milk components [81,82]. MFGM contain around 40% (in weight) of PL, of which 30% is represented by PE, 7% PI, 5% PS, 31% PC, and 20% SM [81,82,83,84]. These phospholipids are differently distributed between the inner and the external layers; PE, PI, and PS are enriched in the internal layer, whereas PC and SM is more abundant in the external layer [81].

Interestingly, phospholipids enriched in milk EVs, such as PC, PS, and SM, have beneficial health effects, due to their roles in maintaining the integrity and functionality of cell membranes and in cell signaling as the messenger in important processes such as differentiation and apoptosis. Consistently, several in vivo and in vitro studies demonstrate that these phospholipids could be used as nutraceutical agents in the adjunct therapy of several pathologies such as cancer, neurodegeneration, and metabolic syndrome [81]. 

## 4. Conclusions

**Figure 1:**
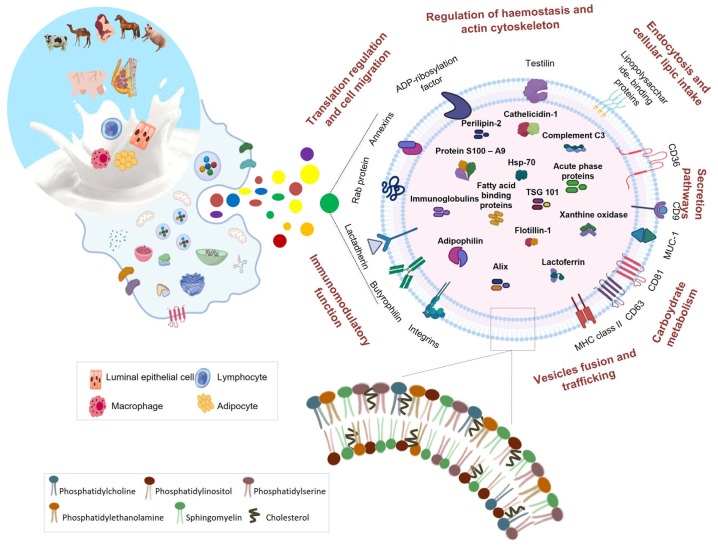
Protein and lipid cargo of milk EVs. Milk EVs are released by different cell types and contain typical milk proteins as well as EV protein markers. EV proteins are involved in different pathways and functional processes. Milk EVs present a lipid composition similar to those of vesicles from other biofluids.

Milk is a complex biological fluid with many protein and lipid species differently distributed within its fractions. The analysis of the protein and lipid cargo of milk EVs is crucial to better define their biogenesis and biological functions, as well as some nutritional aspects of animal milk for human diet. Moreover, a deep knowledge of the lipid and protein composition of EVs present in milk is necessary for understanding the potential use of milk EVs as diagnostic and therapeutic tools. The proteomic characterization of milk EVs revealed that EVs are the milk fraction with the highest protein content and enriched the milk proteome of new protein species, never detected in other fractions. Together with these proteins, milk EVs also contain typical milk proteins that were not previously detected in EVs from other biofluids and the typical protein markers of EVs (i.e., tetraspannins, Tsg 101, Alix) (Figure 1). 

EV protein cargo can be crucial to develop the use of milk EVs in drug delivery as proteins modulate the EV journey throughout body fluids and EV interaction with target cells. Interesting in this contest is the characterization of the glycoproteome of milk EVs, even if the data are still limited to porcine milk, as glycosylation is a parameter strongly affecting EV uptake by target cells.

Another interesting aspect highlighted by the results reported in this review is that the identification of specific tissue/cell proteins confirmed that EVs are released in the milk by different cell types including immune cells. The presence of the typical proteins of immune cells in milk EVs might be related to the plethora of the milk EV-associated biological effects that have been recently reported in the literature. The bioinformatic annotation of EV proteins has also initiated a more comprehensive understanding of the EV functions and cell pathways in which they can be involved (Figure 1).

The comparison between the protein cargo of human and bovine milk EVs evidenced similarities and differences that can be helpful in the formulation of commercial milk or for drug delivery. Indeed, the comparison of milk and colostrum EV proteome revealed that colostrum EVs are significantly enriched in proteins involved in the immune response and that these differences are more accentuated in bovine than in human milk EVs. However, the proteomes of EVs obtained from the milk of other animal species such as donkeys and goats need to be investigated as their milks are more similar to human milk than cow’s milk.

Regarding the lipid content of milk EVs, it is evident that the lipidomic data reported in the literature derive from very few studies. Since the lipid composition of milk differs among different species, it is conceivable that these differences could be mirrored in the lipid composition of milk EVs. However, so far, the few studies have only obtained converging evidence that milk EVs have a lipid composition like that of EVs from other biofluids but different from MFG, as they are enriched in PS and presented low TAG level.

In conclusion, milk EV protein and lipid cargo is of great relevance to address the specific biological properties not only of milk EVs but also of milk. Further studies are needed to understand in more detail the different factors affecting the protein and lipid composition of milk EVs, including diet, health status, and well-being.

## Figures and Tables

**Table 1:** An overview of studies focused on protein and lipid content of milk EVs from different mammal species. The EV isolation protocols and the analytic methods used in the reviewed studies are reported. The terminology used for milk vesicles is based on the reference cited.

Authors	EV Types	Species	Isolation Methods	Analytic Methods	N° Ref.
**proteomics investigation**					
Admyre C, 2007	Vesicles 50 nm (100 K fraction)	Human	Differential centrifugation Sucrose gradient centrifugation	LC-MS/MS	[41]
van Herwijnen M, 2016	EVs	Human	Differential centrifugation; Sucrose gradient centrifugation	LC-MS/MS	[42]
Vaswani K, 2021	Exosomes	Human and Bovine	Differential centrifugation; Exclusion chromatography	LC-MS/MS (IDA Mass Spectrometry)	[43]
Yang M, 2017	Exosomes	Human and Bovine	Differential centrifugation; Sucrose gradient centrifugation; Filtration	LC-MS/MS	[44]
Reinhardt T, 2012	Exosomes 50–100 nm	Bovine	Differential centrifugation; Sucrose gradient centrifugation; Filtration	On line 2D peptide chromatography-MS	[45]
Reinhardt T, 2013	Exosomes	Bovine	Differential centrifugation; Sucrose gradient centrifugation	On line 2D peptide chromatography -MS	[46]
Samuel M, 2017	Exosomes 30–150 nm	Bovine	Differential centrifugation; OptiPrep™ density gradient centrifugation	LC-MS/MS-based label free quantitative proteomics	[47]
Benmoussa A, 2018	Exosomes (100k and 35k fractions)	Bovine	Differential ultracentrifugation; Filtration	LC-MS/MS label free quantification	[48]
Rahman M. 2021	Exosomes 145–150 nm	Bovine	Differential centrifugation; Purification by acetic acid; Sequential filtration	LC-MS/MS	[49]
Rahman M, 2021	small EVs 145–167 nm	Bovine	Differential centrifugation; Purification by acetic acid	Nano-LC-MS/MS	[50]
Chen T, 2017	Exosomes	Porcine	Differential centrifugation; Sucrose gradient centrifugation; Filtration	LC-ESI-MS/MS	[51]
Ferreira R, 2021	Exosomes 100nm	Porcine	Ultracentrifugation Size exclusion chromatography	LC-MS/MS	[52]
Sedykh S, 2017	Exosomes < 30–40 nm	Horse	Sequential ultracentrifugations	2D gel electrophoresis MALDI-TOF	[53]
**lipidomics investigations**					
Chen W, 2021	Exosomes 30–50 nm	Human	Differential centrifugation; Filtration	LC-MS/MS	[54]
Blans K, 2017	EVs 147–189 nm	Human and Bovine	Differential ultracentrifugation;Size-exclusion chromatography	Thin Layer Chromatography	[24]
Grossen P, 2021	EVs 50–150 nm	Bovine	Sequential ultracentrifugation; OptiPrep^TM^ density gradient	MS-Lipotype GmbH	[55]
Yassin M, 2016	Exosomes 50–90 nm	Dromedary	Differential ultracentrifugation	HPLC	[56]

## Data Availability

Not applicable.
